# The BOLD Public Health Response to Alzheimer’s Disease and Related Dementias: Past, Present, and Future

**DOI:** 10.1093/geroni/igaa057.2541

**Published:** 2020-12-16

**Authors:** Lisa McGuire, Nia Reed

## Abstract

Achieving meaningful progress against Alzheimer’s disease and related dementias (ADRD) requires an urgent public health response. Since 2005, the Alzheimer’s Association, Centers for Disease Control and Prevention (CDC), and other public health partners collaborated on the Healthy Brain Initiative (HBI). HBI seeks to advance public health awareness of and action on ADRD as a public health issue. The HBI Road Map Series, State and Local Public Health Partnerships to Address Dementia: The 2018–2023 Road Map (S&L RM) and Road Map for Indian Country, provide the public health with concrete steps to respond to the growing burden of ADRD in communities, consistent with the aim of the Building Our Largest Dementia (BOLD) Infrastructure for Alzheimer’s Act (P.L. 115-406). This series of RMs for state, local, and tribal public health provide flexible menus of actions to address cognitive health, including ADRD, and support for dementia caregivers with population-based approaches. The purpose of this session is to illustrate public health’s role with ADRD—past, present, and future. An overview of the evolution of HBI RM series will be provided (McGuire) and preliminary evaluation data of 24 actions from S&L RM will be presented (French, Shean, Beatty). Holt, Reed, & McGuire will share lessons learned and next steps from CDC’s Regional Action Institutes designed to facilitate implementation of the RM series across the US. The intersection between the RM series and Age-Friendly Public Health Systems will be described as a starting point for public health (Wolfe, French, Shean).

